# Insuficiência Cardíaca Avançada e o Surgimento de Novos Marcadores de Prognóstico: Onde Estamos?

**DOI:** 10.36660/abc.20240453

**Published:** 2024-09-17

**Authors:** Meliza Goi Roscani

**Affiliations:** 1 Universidade Federal de São Carlos Departamento Médico Divisão de Cardiologia São Carlos SP Brasil Divisão de Cardiologia do Departamento Médico da Universidade Federal de São Carlos, São Carlos, SP - Brasil

**Keywords:** Volume Sistólico, Insuficiência Cardíaca, Prognóstico, Biomarcadores

A insuficiência cardíaca (IC) avançada é uma síndrome complexa caracterizada pela presença de sintomas graves e persistentes de IC, disfunção cardíaca importante, episódios de congestão pulmonar ou sistêmica que requerem altas doses de diuréticos intravenosos, episódios de baixo débito que requerem inotrópicos ou drogas vasoativas, arritmias malignas, hospitalização nos últimos 12 meses ou comprometimento grave da capacidade de exercício.^1^ A utilidade de múltiplos marcadores com boa acurácia na detecção de pior prognóstico é cada vez mais explorada na literatura.^2^ O peptídeo natriurético cerebral (BNP) e o NT-proBNP são considerados biomarcadores indicadores prognósticos na IC e podem ajudar a identificar pacientes com maior gravidade da doença.^3^

Recentemente, o aparecimento de novos marcadores ecocardiográficos parece desempenhar um papel importante na detecção de pacientes com progressão mais grave desta síndrome. Vários parâmetros derivados da técnica inovadora de ecocardiografia *speckle tracking* (STE) são de grande valor no diagnóstico e prognóstico de diversas patologias.^4–7^

Tatar et al.^8^ investigaram a associação entre parâmetros de *strain* diastólico, incluindo E/e’ SR medido por STE e resultados de curto prazo em pacientes com IC avançada. Um total de 116 pacientes foram encaminhados para avaliação ecocardiográfica após avaliação inicial no pronto-socorro/ambulatório antes da admissão na unidade de terapia intensiva (UTI)/enfermaria e também antes da terapia diurética. Os pacientes foram acompanhados por um mês e qualquer reinternação por piora dos sintomas de IC e qualquer mortalidade foi registrada. Durante o seguimento, 16 pacientes morreram e o nível sérico de proBNP e E/e’ SR foram preditores independentes de mortalidade. E/e’ SR apresentou sensibilidade e especificidade de 86,7 e 58,0% para predizer mortalidade. A grande relevância deste estudo é que ele foi, até onde sabemos, único em pacientes com ICFEr avançada e o momento da avaliação ecocardiográfica dentro de 24 horas após a admissão também foi muito preciso.

Para melhor compreender esses achados, a função diastólica do ventrículo esquerdo (VE) é influenciada pela sobrecarga atrial esquerda, interação entre ventrículo direito (VD) e VE, contenção pericárdica, função sistólica do VE, dessincronia da função do VE e VD, fluxo sanguíneo coronariano e perfusão tecidual. A avaliação do relaxamento do VE e da pressão de enchimento do VE pela ecocardiografia em pacientes com IC é potencialmente útil na identificação de pacientes com maior gravidade desta síndrome.^9^

A taxa de *strain* diastólico pode ser afetada pela pressão do átrio esquerdo, pelo relaxamento contínuo do VE e pela rigidez do VE e pode estar bem correlacionada com a pressão de enchimento do VE e a pressão de oclusão capilar pulmonar.^10^

**Figura 1:**
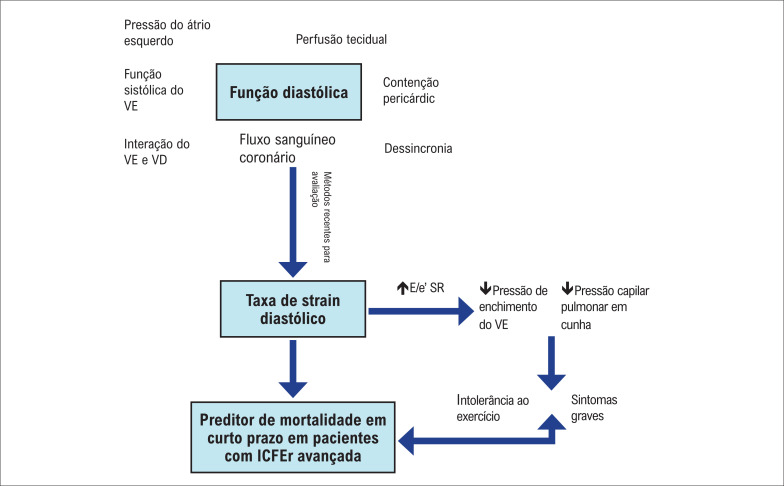
Fluxograma do papel da taxa de strain diastólico no prognóstico de pacientes com insuficiência cardíaca avançada. VE: ventrículo esquerdo; VD: ventrículo direito; E/e’SR: relação entre a velocidade de enchimento transmitral precoce e a taxa de strain diastólico precoce; ICFER: insuficiência cardíaca com fração de ejeção reduzida.

Considerando que pacientes com IC avançada podem evoluir com função diastólica gravemente comprometida, que está associada à intolerância ao exercício e sintomas mais graves, valores mais elevados de E/e’ SR podem discriminar pacientes com desfechos desfavoráveis em curto prazo, incluindo mortalidade durante o acompanhamento de 1 mês. Isso está bem representado na Figura 1.

Embora a E/e’SR seja considerada um método inovador, algumas limitações devem ser descritas, incluindo a técnica de aquisição de imagens, falta de padronização para medidas de SR diastólica e menor precisão com taquicardia.^9,11^

O estudo de Tatar et al.^8^ corroborado com outros estudos que investigaram a taxa de *strain* diastólico sobre desfechos adversos em pacientes com IC. He et al.^12^ descobriram que a taxa de *strain* longitudinal diastólico precoce global do VE obtida a partir do rastreamento da ressonância magnética cardiovascular foi independentemente associada a resultados adversos em pacientes com IC com fração de ejeção preservada. Hortegal et al.^13^ resumiram o papel do STE na detecção de pacientes com IC preservada, reforçando a importância de novos métodos de avaliação e prognóstico.

Em resumo, métodos ecocardiográficos mais elegantes estão surgindo para melhor investigar a função diastólica em pacientes com IC e podem desempenhar um valor prognóstico significativo com boa precisão na detecção de pacientes com resultados desfavoráveis a curto e longo prazo.
