# Supplementary material for: Insuficiência Cardíaca Avançada e o Surgimento de Novos Marcadores de Prognóstico: Onde Estamos?
Source: Arq Bras Cardiol. 2024 Sep 17;121(8):e20240453. [Article in Portuguese] doi: 10.36660/abc.20240453 (PMC11495803; doi:10.36660/abc.20240453)
Supplement: Translation

# Translation: Advanced Heart Failure and Onset of New Prognostic Markers: Where are We?

**DOI:** 10.36660/abc.20240453i

**Authors:** Meliza Goi Roscani

**Affiliations:** 1 São Carlos SP Brazil Divisão de Cardiologia do Departamento Médico da Universidade Federal de São Carlos, São Carlos, SP – Brazil

**Keywords:** Stroke Volume, Heart Failure, Prognosis, Biomarkers

Advanced heart failure (HF) is a complex syndrome characterized by the presence of severe and persistent symptoms of HF, important cardiac dysfunction, episodes of pulmonary or systemic congestion requiring high-dose of intravenous diuretics, episodes of low output requiring inotropes or vasoactive drugs, malignant arrhythmias, hospitalization in the past 12 months or severe impairment of exercise capacity.^1^ The utility of multiple markers that have good accuracy in the detection of worse prognosis are increasingly being explored in the literature.^2^ Brain natriuretic peptide (BNP) and NT-proBNP are considered biomarkers prognostic indicators in HF and can help to identify patients with more severity of disease.^3^

Recently, the onset of new echocardiographic markers appears to play an important role in detecting patients with more severe progression of this syndrome. Various parameters derived from the innovative speckle tracking echocardiography (STE) technique are of great value in the diagnostic and prognostic of several pathologies.^4–7^

Tatar et al.^8^ investigated the association between diastolic strain parameters including E/e’ SR measured by STE and short-term outcomes in advanced HF patients. A total of 116 patients were referred to the echocardiographic assessment following their initial evaluation in the emergency room/outpatient clinic before admission to the intensive care unit (ICU)/ward and also before diuretic therapy. Patients were followed for one month and any re-hospitalization due to worsening of HF symptoms and any mortality was recorded. During the follow-up, 16 patients died and serum proBNP level and E/e’ SR were independent predictors of mortality. E/e’ SR had a sensitivity and specificity of 86.7 and 58.0% for predicting mortality. The great relevance of this study is that this was, to the best of our knowledge, unique in advanced HFrEF patients and the timing of echocardiographic assessment within 24 hours of admission was also very accurate.

To better understand these findings, left ventricular (LV) diastolic function is influenced by left atrial overload, right ventricular (RV) and LV interaction, pericardial contention, LV systolic function, desynchrony of LV and RV function, coronary blood flow, and tissue perfusion. Assessment of LV relaxation and LV filling pressure by echocardiography in HF patients is potentially useful in identifying patients with more severity of this syndrome.^9^

The diastolic strain rate may be affected by left atrium pressure, ongoing LV relaxation, and LV stiffness and can be well correlated with LV filling pressure and pulmonary capillary wedge pressure.^10^

Considering that advanced HF patients may course with severely impaired diastolic function, which is associated with exercise intolerance and more severe symptoms, higher E/e’ SR values may discriminate patients with short-term unfavorable outcomes, including mortality during the 1-month follow-up. This is well represented in Figure 1.

**Figure 1:** Flowchart of the role of diastolic strain rate on the prognosis of advanced heart failure patients. LV: left ventricle; RV: right ventricle; E/e’SR: the ratio of transmitral early filling velocity to early diastolic strain rate; HFrEF: heart failure with reduced ejection fraction.

Although E/e’ SR is considered an innovative method, some limitations should be described, including technical image acquisition, lack of standardization for diastolic SR measurements, and lower precision with tachycardia.^9,11^

The study of Tatar et al.^8^ corroborated with other studies that investigated diastolic strain rate on adverse outcomes in HF patients. He et al.^12^ found that LV global early diastolic longitudinal strain rate obtained from cardiovascular magnetic resonance feature tracking was independently associated with adverse outcomes in patients with HF with preserved ejection fraction. Hortegal et al.^13^ summarized the role of STE in detecting patients with preserved HF, reinforcing the importance of new assessment and prognosis methods.

In summary, more elegant echocardiographic methods are emerging to better investigate diastolic function in patients with HF and may play a significative prognostic value with good accuracy in detecting patients with unfavorable short- and long-term outcomes.
